# Asian infants show preference for own-race but not other-race female faces: the role of infant caregiving arrangements

**DOI:** 10.3389/fpsyg.2015.00593

**Published:** 2015-05-07

**Authors:** Shaoying Liu, Naiqi G. Xiao, Paul C. Quinn, Dandan Zhu, Liezhong Ge, Olivier Pascalis, Kang Lee

**Affiliations:** ^1^Zhejiang Sci-Tech University, Hangzhou, China; ^2^University of Toronto, Toronto, ON, Canada; ^3^University of Delaware, Newark, DE, USA; ^4^Laboratoire de Psychologie et Neurocognition – Université Grenoble Alpes, Centre National de la Recherche Scientifique, Grenoble, France

**Keywords:** infant, gender preference, caregiving arrangements, other-race effect, age-related

## Abstract

Previous studies have reported that 3- to 4-month-olds show a visual preference for faces of the same gender as their primary caregiver (e.g., Quinn et al., 2002). In addition, this gender preference has been observed for own-race faces, but not for other-race faces (Quinn et al., 2008). However, most of the studies of face gender preference have focused on infants at 3–4 months. Development of gender preference in later infancy is still unclear. Moreover, all of these studies were conducted with Caucasian infants from Western countries. It is thus unknown whether a gender preference that is limited to own-race faces can be generalized to infants from other racial groups and different cultures with distinct caregiving practices. The current study investigated the face gender preferences of Asian infants presented with male versus female face pairs from Asian and Caucasian races at 3, 6, and 9 months and the role of caregiving arrangements in eliciting those preferences. The results showed an own-race female face preference in 3- and 6-month-olds, but not in 9-month-olds. Moreover, the downturn in the female face preference correlated with the cumulative male face experience obtained in caregiving practices. In contrast, no gender preference or correlation between gender preference and face experience was found for other-race Caucasian faces at any age. The data indicate that the face gender preference is not specifically rooted in Western cultural caregiving practices. In addition, the race dependency of the effect previously observed for Caucasian infants reared by Caucasian caregivers looking at Caucasian but not Asian faces extends to Asian infants reared by Asian caregivers looking at Asian but not Caucasian faces. The findings also provide additional support for an experiential basis for the gender preference, and in particular suggest that cumulative male face experience plays a role in inducing a downturn in the preference in older infants.

## Introduction

The development of face processing is greatly shaped by visual experience (Le Grand et al., 2001; Pascalis et al., 2002, 2005; Kelly et al., 2007a,b; Cassia et al., 2009; see Lee et al., 2013). Previous studies have reported that differential face gender experience provided by caregiving interactions influences the formation of face gender categories at a very early stage of life (Quinn et al., 2002, 2008, 2010; Rennels and Davis, 2008). Infants tend to prefer the face gender of their primary caregiver. Due to the fact that previous studies only focused on face gender preference before 4 months of age, it is unclear how caregiving practices affect face gender preference in later infancy (cf. Quinn, 2002). To bridge this gap in the literature, the current study investigated the influence of caregiving involvements on face gender preference in 3-, 6-, and 9-month-old infants. Moreover, all of the previous studies examined the face gender preferences of Caucasian infants from Western countries in Europe or North America. It is therefore unclear whether the female face preference can be extended to infants from other racial groups and different cultures with distinct child rearing practices. The present study specifically recruited Chinese infants to examine the role of Asian caregiving arrangements in the development of the female face preference.

Extensive studies suggest that face experience plays an important role in shaping the development of face processing in infancy (e.g., Pascalis et al., 2002; Kelly et al., 2007a,b, 2009). Among the various kinds of face experience, the face interactions provided by caregivers are one of the earliest face experiences that infants obtain. As indicated by recent studies, infants spend approximately 70% of their time with female faces, inclusive of an approximate 50% contribution from a female caregiver and an approximate 20% contribution from other female faces, as estimated by parental reports during the first year of life (Rennels and Davis, 2008). The 70% estimate of female face experience has been corroborated by data obtained with a head mounted camera during the first 3 months of life (Sugden et al., 2014). Most relevant to the present paper is the finding that how infants represent gender information in faces is influenced by the gender of the primary caregiver by 3–4 months of age. Quinn et al. (2002) presented infants with male versus female face pairings and reported that the infants who were primarily raised by female caregivers preferred the female faces. In contrast, infants raised mostly by male caregivers showed a reliable preference for male faces. Also, consistent with an experiential account, a more recent study found that newborns did not show any gender preference, and that the female face preference is limited to own-race faces at 3 months of age (Quinn et al., 2008). In addition, Quinn et al. (2010) found 3- to 4-month-olds were able to generalize their female face preference across age and display a preference for a girl over boy prototype face. Taken together, the results suggest that differential female versus male face experience in caregiving contributes to the differences in how infants respond to female versus male faces.

According to Quinn et al. (2002), the face gender preferences of infants reflect caregiving arrangements, which are determined by differential involvements of female and male caregivers. This account implies that day-to-day caregiving interactions play a crucial role in how infants come to represent face gender information. Extensive experience with a female or male primary caregiver helps infants form a representation of face gender that favors the gender of the more frequently experienced caregiver at a relatively early stage of life (i.e., 3–4 months). Some prior studies suggest that face gender categories formed in early infancy undergo further development during later infancy (e.g., 7–10 months, Leinbach and Fagot, 1993; Younger and Fearing, 1999). However, it is presently unclear how caregiving practices influence responsiveness to face gender categories over 4 months of age.

The female face preference suggests that the female face representation is formed earlier than the male face representation. Studying age related development in face gender preference offers an extension of the current literature, and can provide evidence relevant to how gender representations may change in the first year of life. One possible developmental model is a ratio or proportional experience model. By this model, a consistent difference between the female and male caregiving involvement could result in a constant reliable difference in female and male face representation, and we would expect a comparable female face preference across ages. Another possible developmental model is a threshold model. That is, when male experience reaches some threshold amount, we may observe a decreased female face preference with age. This is because with increased age, infants might eventually acquire sufficient male face experience to form a representation for male faces comparable to that of female faces. The later formed male face representation would presumably interfere with the initial female face preference, which was driven by the earlier-formed female representation. If the latter is the case, we may find a female face preference in the younger infant participants in our sample. By contrast, the older infants in our sample may not show a differential preference for female or male faces.

The present study was designed to examine the influence of caregiving on the face gender preference during infancy. Specifically, it was conducted with Chinese infants at 3, 6, and 9 months of age, who were primarily reared by female caregivers. Like caregiving in North America, caregiving in China is female dominant. In addition, the relative proportions of female and male involvement in infant’s caregiving are relatively stable in Asian countries. China thus offers an ideal environment to study the influence of caregiving on the development of face gender representation in the first year of life.

We used a visual preference task to examine the face gender preferences of infants. In addition, we investigated each infant participant’s caregiving arrangement, as determined by the amount of female and male involvement in caregiving. The caregiving arrangement allows us to directly examine the relationship between an infant’s face gender preference and the involvement of female and male caregivers.

There is one additional rationale for the current study. To our knowledge, all of the prior studies on gender preference were performed with predominantly Caucasian participants. This makes it unclear whether the finding of Quinn et al. (2008) that the female face preference is limited to own-race faces indicates that the face gender preference is limited to faces from the infant’s own race group (i.e., own-race faces) or to a specific type of faces (i.e., Caucasian faces). The present study for the first time investigated whether the female face preference can be observed in Asian infants, and whether it will hold for Asian faces, but not for Caucasian faces. We therefore used both own-race Asian faces and other-race Caucasian faces to examine the face gender preferences of Asian infants. If the face gender preference is limited to own-race faces, we should observe it only in own-race Asian faces, but not in other-race Caucasian faces.

## Materials and Methods

### Participants

We recruited infant participants through advertisements posted on community news boards. Eighty-four Chinese infants participated in the current study, and their parents gave consent for the infants to participate in the current study. There were 11 participants in the 3-month-old group (age range = 86–110 days; 4 females, 7 males), 40 participants in the 6-month-old group (age range = 178–196 days; 15 females, 25 males), and 33 participants in the 9-month-old group (age range = 268–289 days; 17 females, 16 males). The relatively small sample size in the 3-month-old group was partially due to the fact that several previous gender preference studies have reported a robust gender preference with small samples of 3-month-olds (e.g., Quinn et al., 2002). By contrast, the older age groups have not previously been tested for gender preference. We therefore needed stronger statistical power to evaluate the performance of these groups.

All participants were healthy, full-term infants. Thirteen additional infants also participated in the current study, but were excluded from final analysis because of extreme side bias (*n*_3-month_ = 6; *n*_6-month_ = 6) or fussiness (*n*_9-month_ = 1). Extreme side bias occurred when a participant spent more than 90% of their looking time on one side of the display for each presentation. Based on parental report, no infants had direct other-race face experience before the current study.

### Materials

**FIGURE 1:**
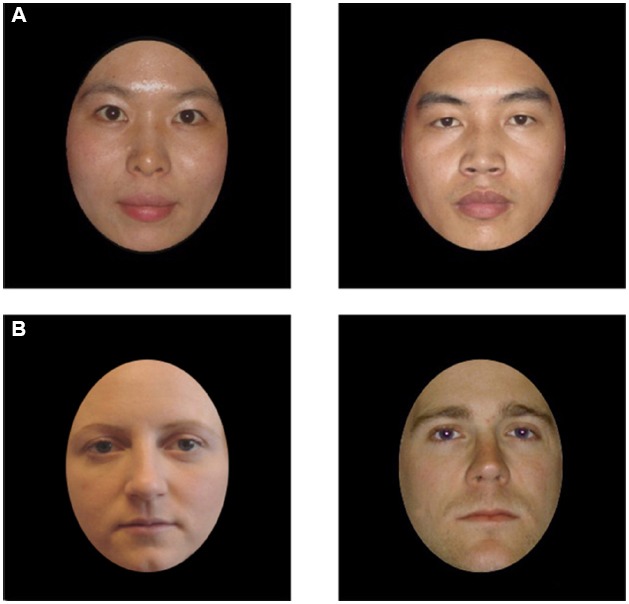
**Examples of a female–male own-race Asian face pair (A) and a female–male other-race Caucasian face pair (B)**.

The stimuli were face pairs comprised of female and male frontal-view images placed side-by-side. Examples can be observed in Figure 1. Facial external features were removed by an oval mask, which was sized 13.80 cm in width and 19.07 cm in height. We made further adjustments to make sure that all faces were of the same size and comparable in brightness and contrast. The faces were between 25 and 29 years of age. Overall, three pairs of Asian faces and three pairs of Caucasian faces were used. Eight Chinese adults who were blind to the purpose of the research were recruited to rate each face’s attractiveness and gender distinctiveness using Likert scales. The results showed that these faces were comparable in attractiveness (*p* = 0.874) and gender distinctiveness (*p* = 0.723).

### Procedure

All infants were tested in a quiet room while sitting on the lap of a parent. A 42-inch monitor (width = 90.00 cm, height = 57.00 cm) was placed in front of participants for a viewing distance of approximately 75 cm. Before the test started, an experimenter examined the position of the infants to make sure they were aligned to the center of the monitor. The parent who held the infant was asked to keep the infant stable during the test. The parent was also asked to keep their eyes closed during testing.

Once a participant was in place, the test started. For the own-race condition, participants would see one own-race female and one own-race male face displayed on each side of the monitor. The two faces were displayed at the eye level of the participants, and were separated by a 33.04 cm (24.85° in visual angle) gap. The face pair was presented cumulatively for 10 s. Then, the left–right positions of the two faces were switched and the faces were presented for another 10 s. Stimulus presentation was controlled by a computer program. The other-race condition was the same as the own-race condition, except that two Caucasian faces were presented. Looking time of the participants was recorded by a camera placed above the monitor. These recordings were used for offline coding to indicate each infant’s proportional looking time for female versus male faces. One of the three female–male face pairs was randomly selected for each participant in each condition. The order of presentation of the races was counterbalanced across infants. The initial position (i.e., the left or right side) of the female face was randomly selected by the computer program.

Each infant’s looking time for faces on the left and right sides was measured by examining each frame of the camera recordings. The camera recordings contained only each participant’s face, without any cues of the screen content that participants were watching. Each video was trimmed according to face pair onset and offset time. We extracted each frame of the video (25 frames/second) to save as an image file. We then randomly presented these images to raters, who determined which side of the screen participants looked at. We finally calculated the numbers of left looking frames and right looking frames for each participant in the own- and other-race conditions, and these were used for calculating percentage looking time. Two independent raters participated in the coding process, and their inter-rater agreement was high (*Pearson r* = 0.90). Therefore, we averaged the looking time measurement from the two raters for the following analyses. A preliminary analysis revealed no significant gender difference for participants; thus, data were collapsed across participant gender in subsequent analyses.

In addition to the face gender preference test, parents completed a questionnaire to report the monthly involvement of each family member in caregiving. The family members included the mother, father, grandmother on the mother’s side, grandmother on the father’s side, grandfather on the mother’s side, and grandfather on the father’s side. For each family member, one of four involvement levels was requested: (1) never, (2) occasionally, (3) often, and (4) always. Parents were asked to report the involvement in every month after the infant’s birth. For example, a 3-month-old’s parents should provide the involvement in the 1st, 2nd, and 3rd month, while a 6-month-old’s parents should provide the involvement from the 1st to the 6th month.

### Chinese Caregiving Arrangements

**FIGURE 2:**
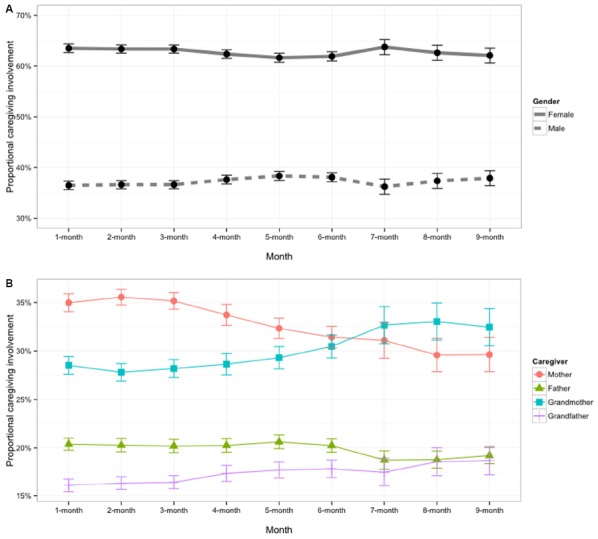
**The proportional caregiving involvement of female and male caregivers (A) and the proportional caregiving involvement of each type of caregiver (i.e., mother, father, grandmother, and grandfather) (B) within each month.** Error bars represents one standard error.

To derive the amount of female and male caregiver involvement for each participant, we translated the involvement responses into proportional involvement scores. To do so, we first converted parent responses into numbers (never = 0, occasionally = 1, often = 2, and always = 3). Based on the caregiving data provided by the parents and in accord with Chinese culture, caregiving provided by grandparents on the mother and father sides was coordinated and offered in alternate months. Thus, for example if the grandparents on the mother side provided caregiving in the first month, then the grandparents on the father side provided caregiving in the second month, and so on. We therefore used a single grandmother score and a single grandfather score in any given month to indicate the involvements of grandparents on the mother and father side. Then, a family involvement score was derived from the sum of each caregiver’s involvement (I_family_ = I_mother_ + I_father_ + I_grandmother_ + I_grandfather_). We calculated female involvement scores by summing scores of mother and grandmother (I_female_ = I_mother_ + I_grandmother_) and male involvement scores by adding up scores of father and grandfather (I_male_ = I_father_ + I_grandfather_). To derive proportional involvement scores for females, we further divided female and male scores by the total family involvement score (ProI_female_ = I_female_/I_family_). On average, females played the majority part in caregiving (63.86%), which did not vary across months [ANOVA, *F*(8, 626) = 0.66, *p* = 0.729]. In addition, we further calculated the proportional involvement scores for each category of caregiver (i.e., mother, father, grandmother, and grandfather). The monthly proportional caregiving involvements are plotted in Figure 2, broken down by female versus male in the top panel, and the more specific caregiving categories in the bottom panel. Overall, Figure 2 illustrates the consistent advantage in female caregiving across the first 9 months of infancy, and provides evidence that the female advantage holds whether one is comparing mother with father or grandmother with grandfather.

## Results

### Face Gender Preference for Own- and Other-Race Faces

We first calculated a female face preference for each participant. This percentage preference score was derived by dividing the female face looking time by the total looking time on the female and male faces and then multiplying that fraction by 100.

To examine the effect of face race and participant age on the female face preference, a 2 (face race: own and other) × 3 (age group: 3, 6, and 9 months) mixed repeated measures analysis of variance (ANOVA) was conducted. We found a significant main effect of race, *F*(1, 81) = 9.25, *p* = 0.003, ${\text{η}}_{\text{p}}^{\text{2}}$ = 0.85. On average, infants showed a significantly larger female face preference for own-race faces (*M* = 57.27%, SD = 17.63%) than for other-race faces (*M* = 46.45%, SD = 21.62%). There was no significant age group effect [*F*(2, 81) = 0.24, *p* = 0.789, ${\text{η}}_{\text{p}}^{\text{2}}$ = 0.09], or interaction between race and age [*F*(2, 81) = 1.59, *p* = 0.210, ${\text{η}}_{\text{p}}^{\text{2}}$ = 0.33].

**FIGURE 3:**
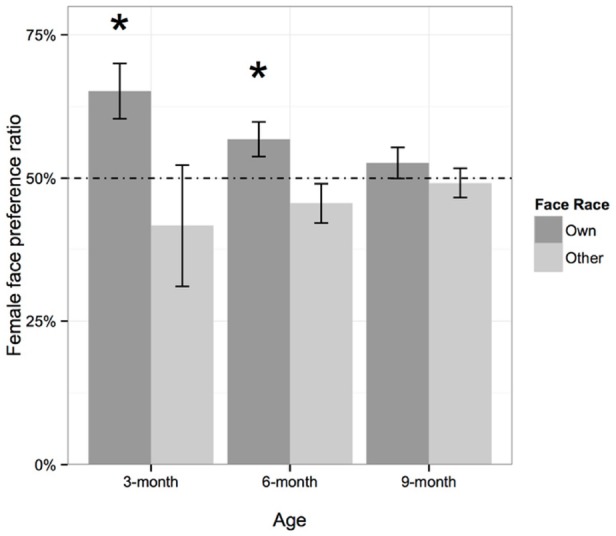
**Mean proportional female face looking time for own- and other-race faces.** Error bars represent one standard error. The asterisks suggest a significant preference for female over male faces (*p < 0.05).

To further examine whether the female face preference at each age was above chance, we performed one-sample *t*-tests for each age group, in which the female face preference was compared to chance responding (i.e., 50%). For own-race faces, as shown in Figure 3, we found that 3- and 6-month-olds showed a significant female face preference [3-month-olds: *M* = 65.18%, SD = 15.94%, *t*(10) = 3.16, *Cohen’s d* = 2.00, *p* = 0.010; 6-month-olds: *M* = 56.79%, SD = 19.07%, *t*(39) = 2.25, *Cohen’s d* = 0.72, *p* = 0.030]. However, 9-month-olds did not show a preference for female or male faces [*M* = 52.67%, SD = 15.57%, *t*(32) = 0.98, *Cohen’s d* = 0.35, *p* = 0.332]. Moreover, a Pearson correlation analysis revealed that the female face preference decreased with age [*r*(82) = –0.208, *p* = 0.029]. As for the other-race Caucasian faces, we did not find a gender preference at any age: 3-month-olds: *M* = 41.67%, SD = 35.20%, *t*(10) = –0.77, *Cohen’s d* = –0.50, *p* = 0.451; 6-month-olds: *M* = 45.56%, SD = 21.90%, *t*(39) = –1.28, *Cohen’s d* = –0.41, *p* = 0.207; 9-month-olds: *M* = 49.13%, SD = 14.99%, *t*(32) = –0.33, *Cohen’s d* = –0.11, *p* = 0.742.

Taken together, the present results regarding the female face preference replicated previous findings that infants at 3 months of age show a reliable preference for own-race female faces over own-race male faces (Quinn et al., 2002, 2008; Hillairet de Boisferon et al., 2014). Also, the current findings indicate that the female face preference is present only in 3- and 6-month-olds. The 9-month-olds spent equivalent amounts of time looking at female versus male faces. When we consider the downturn in the female face preference with age coupled with the relative constancy of the female advantage in caregiving, the two outcomes taken together suggest that infants might gradually gain sufficient male face experience to form comparable representations of female and male faces, thereby significantly reducing their visual preference for female faces, a point that we explore further in the next section of the Results.

In addition, in accord with Quinn et al. (2008), the female face preference was only observed in own-race faces, but not in other-race faces. This finding indicates that the findings of Quinn et al. (2008) are not limited to Caucasian participants or Caucasian faces. Rather, the results seem to reflect a general phenomenon regarding difference in access to gender diagnostic information in own- versus other-race faces.

### The Role of Cumulative Male Caregiving Involvement in Face Gender Preference

**FIGURE 4:**
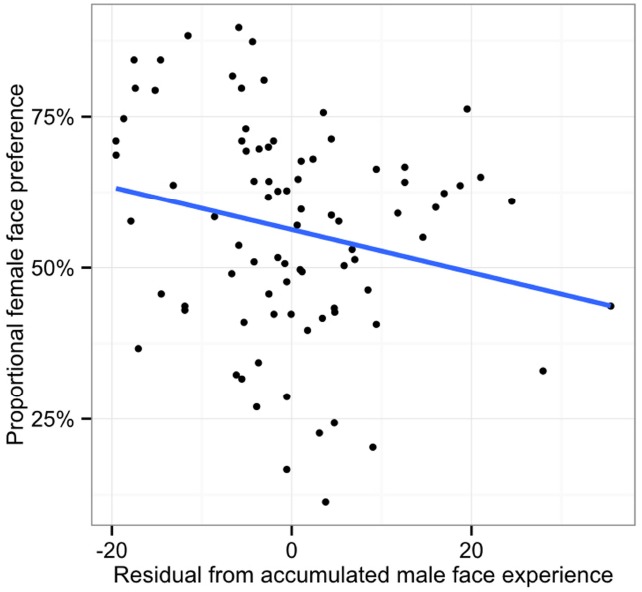
**Linear correlation between the accumulated male face experience and the proportional own-race female face preference after controlling for the effect of the female–male face experience ratio**.

To examine the contribution of accumulated male face experience to the downturn in the female face preference, we first calculated the cumulative male face experience by taking the data in Figure 2 and summing each participant’s male face experience across months. Not surprisingly, cumulative male face experience increased with age [*r*(82) = 0.52, *p* < 0.001]. We then performed a partial Pearson correlation between the accumulated male face experiences gained in caregiving and the proportional female face preference after controlling for the effect of the proportional female face experience. Importantly, the results indicated that the amount of male face experience was negatively correlated with the female face preference [partial *r*(81) = –0.22, *p* = 0.046, Figure 4], when the influence of female–male experience ratio was controlled. The pattern of correlations suggests that with increased age, infants may obtain sufficient male experience to account for the downturn in the female face preference. Additionally, we failed to find such partial correlation between the male caregiver experience and the other-race Caucasian female face preference, partial *r*(81) = 0.16, *p* = 0.138. The results indicate that male experience in caregiving only influenced infant’s own-race face gender preference, but not other-race face gender preference.

## Discussion

The present study investigated face gender preference in 3-, 6-, and 9-month-old Chinese infants who were primarily reared by female caregivers. Three major findings were obtained: (1) Three- and 6-month-olds exhibited a significant preference for female own-race faces over male own-race faces. No such gender preference was revealed in 9-month-olds. (2) The female face preference decreased with the increase in cumulative male face experience. (3) Infants failed to show any gender preference for other-race Caucasian faces at any age. These results together indicate the important role of face gender experience within caregiving practice in how infants respond to face gender categories.

The finding of a female face preference in 3-month-olds is consistent with previous findings that 3- to 4-month-old Caucasian infants preferred own-race Caucasian female faces over male faces (Quinn et al., 2002, 2008, 2010; Hillairet de Boisferon et al., 2014). Moreover, by examining female and male caregiving involvement, we found a caregiving bias in favor of females in Asian culture similar to that previously reported in Western culture, in which females form the category of greater experience (Rennels and Davis, 2008; Sugden et al., 2014). The data from the three studies taken together suggest female face dominance in infant face experience. Moreover, the fact that the current study and that of Rennels and Davis (2008) relied on parental report data, whereas Sugden et al. (2014) reported data obtained with head mounted cameras, suggests that an approximate 2 to 1 female to male ratio is independent of the particular measure of experience. In addition, the fact that the prior studies were conducted in North America, one in the US (Rennels and Davis, 2008) and one in Canada (Sugden et al., 2014), whereas the current study was conducted in China, suggests that the dominant female to male face experience transcends culturally-based differences in Western and Eastern child rearing practices. The relatively rich interaction with female caregivers contributes to forming a more robust representation of female faces relative to that of male faces at an early stage of life, and has even been shown to have specific neural correlates (Righi et al., 2014). The discrepancy in the robustness of the representations based on differential experience presumably leads to a preference for female faces, which has been shown to generalize even to a girl prototype face (Quinn et al., 2010).

In addition to the female face preference found in the 3-month-olds, the present study also examined the gender preference in older infants. Despite the fact that female proportional involvement in caregiving remains stable across the first 9 months of age, we only observed the female face preference in the 3- and 6-month-olds. No significant gender preference was found in the 9-month-olds. Moreover, as indicated by the correlation analysis, the female face preference decreased significantly with the increase in accumulated male face experience. This overall pattern of results is consistent with an account in which a representation of female faces is formed relatively early because of the greater female caregiving experience. Representation of male faces, due to the lesser male face experience, may take longer to develop. The quality of the representation of male faces may eventually reach the same level as the quality of the representation for female faces, and then eventuate in a null preference between female and male faces in older infants. The finding of a developmental downturn in face gender preference supports the hypothesis that a face gender representation is developing with face experience until the amount of experience reaches a threshold amount. Such a mechanism would account for the age-related female preference decrease.

The age-related change in the female face preference is broadly consistent with other changes in the development of face representation more generally. The emergence of face prototypes was reported around 3 months of age (de Haan et al., 2001). Evidence for categorical differentiation of faces can be found in the preference for the more familiar gender (Quinn et al., 2002) and race (Kelly et al., 2005) at 3–4 months of age. This preliminary representation of social categories may undergo further development as the infant gains more experience, which may occur around 7–10 months of age. Infants at this age start to exhibit more reliable abilities to categorize face gender and race (Leinbach and Fagot, 1993; Younger and Fearing, 1999; Anzures et al., 2010). At these older ages, the preference found in young infants may be likely to disappear, which reflects comparable efficiency in processing faces of greater or lesser experience (de Haan and Nelson, 1999) or a transition phase for further development (Liu et al., 2015). Moreover, representation of social categories in the changeover from infancy to early childhood might follow a perceptual-to-conceptual transition (Madole and Oakes, 1999; Quinn et al., 2001, 2013). For example, 3-year-olds tend to choose a novel object from a person of their own gender (Shutts et al., 2010). Five-year-olds show a similar race-based social preference for own-race individuals over other-race ones (Kinzler and Spelke, 2011). It would be worthwhile in future studies to bridge the gap between the face biases of infants and social biases of children so as to reveal a fuller trajectory in the development of responsiveness to the social world.

As for the other-race Caucasian faces, we did not find any preference for female or male faces at any age. This finding supports the argument that it is face race (i.e., own-race vs. other-race) rather than a specific type of face (e.g., Asian faces or Caucasian faces) that leads to the female face preference being observed only for own-race faces. This race-related difference may indicate that the extensive experience with own-race faces influences the perceptual cues used for categorizing face gender. With extensive experience with own-race faces, infants might develop a race specific strategy for processing facial gender information by relying on the gender diagnostic information specific to own-race faces. However, the cues used to process gender in own-race faces might not apply to the processing of gender in other-race faces, which results in the disappearance of the gender preference for other-race faces. This perceptual argument is supported by a recent study with Caucasian infants, which reported that hairline is a diagnostic cue for processing the gender of Caucasian faces (Hillairet de Boisferon et al., 2014). However, this cue could not apply for processing the gender of the Asian faces in the present study, given that the hairline and other external facial features were removed.

The race specific female face preference suggests a hierarchical structure in representing own-race faces, in which race is superordinate to gender information. The present findings, along with those of Quinn et al. (2008), are additionally consistent with several adult findings indicating that face gender information in own-race faces is processed more readily relative to that in other-race faces (e.g., O’Toole et al., 1996; Rossion, 2002). Together with the findings that the processing of face race information by infants was not affected by face gender (Kelly et al., 2005, 2007a,b, 2009), the pattern of outcomes taken together supports a model of face processing, in which face race information may be processed before gender information. This model, which is further supported by event related potential (ERP) evidence in which the face race processing related ERP components can be observed earlier than components associated with gender processing (Ito and Urland, 2003), challenges the classic face processing model that race and gender are processed independently (Bruce and Young, 1986).

We acknowledge a few limitations in the current study. It only investigated face experience within the context of family caregiving practices. However, infants, especially older ones, also acquire face experience outside the family environment, which could potentially affect the development of gender categories. For example, in Rennels and Davis (2008), the ratio of non-caregiver female faces to non-caregiver male faces was more than 2 to 1, which is consistent with the possibility that female caregivers may have more same-sex friends who come to interact with the infant. To overcome this limitation, future studies could consider using other investigative techniques, such as head-mounted cameras to record the infant’s face experience both inside and outside the family caregiving environment (Sugden et al., 2014). In addition, it is not completely clear whether disproportional gender experience within caregiving contributes to the development of other aspects of face processing. Although Quinn et al. (2002) showed that infants are more likely to represent a set of female faces as individual exemplars and a set of male faces at the category level of male, we do not know if perceptual narrowing occurs in the processing of gender. For example, it is possible that infants might develop more sophisticated capabilities to recognize faces (Kelly et al., 2007b, 2009) or to process facial expressions (Kahana-Kalman and Walker-Andrews, 2001) specific to their more familiar gender category. Future studies might consider using multiple tasks to further investigate the role of face experience for a variety of aspects of face processing.

### Conflict of Interest Statement

The authors declare that the research was conducted in the absence of any commercial or financial relationships that could be construed as a potential conflict of interest.
